# Complete mitochondrial genome of the gray reef shark, *Carcharhinus amblyrhynchos* (Carcharhiniformes: Carcharhinidae)

**DOI:** 10.1080/23802359.2020.1765208

**Published:** 2020-05-14

**Authors:** Nicholas Dunn, Shaili Johri, David Curnick, Chris Carbone, Elizabeth A. Dinsdale, Taylor K. Chapple, Barbara A. Block, Vincent Savolainen

**Affiliations:** a Institute of Zoology, Zoological Society of London, London, UK; b Department of Life Sciences, Imperial College London, Ascot, UK; c Hopkins Marine Station, Stanford University, Pacific Grove, CA, USA; d Department of Biology, San Diego State University, San Diego, CA, USA; e Coastal Oregon Marine Experiment Station, Oregon State University, Newport, OR, USA

**Keywords:** Shark, British Indian Ocean Territory, *Carcharhinus amblyrhynchos*, mitochondrial genome

## Abstract

We report the first mitochondrial genome sequences for the gray reef shark, *Carcharhinus amblyrhynchos*. Two specimens from the British Indian Ocean Territory were sequenced independently using two different next generation sequencing methods, namely short read sequencing on the Illumina HiSeq and long read sequencing on the Oxford Nanopore Technologies’ MinION sequencer. The two sequences are 99.9% identical and are 16,705 base pairs (bp) and 16,706 bp in length. The mitogenome contains 22 tRNA genes, two rRNA genes, 13 protein-coding genes and two non-coding regions; the control region and the origin of light-strand replication (OL).

## Main text

The gray reef shark *Carcharhinus amblyrhynchos,* is a highly-social, reef-dependent species distributed widely in the tropical Indo-Pacific and currently listed as ‘Near Threatened’ in the IUCN Red List (Smale 2009). Populations have declined due to illegal fishing activities (Osgood and Baum 2015; Ferretti et al. 2018). Whilst there have been genetic studies conducted on the species (Holmes et al. 2009; Momigliano et al. 2015, 2017), its mitogenome has not yet been described.

We describe the complete mitochondrial genome of *C. amblyrhynchos*. Tissue was sampled as fin clips from two specimens in the British Indian Ocean Territory in March 2018. Specimen 1 (GenBank MT093205) was a female tagged at location −5.46386° 71.77841° and specimen 2 (GenBank MT104515) was a male tagged at −5.24956° 71.79906°. Samples were stored at Hopkins Marine Station before specimen 1’s tissue was transferred to Silwood Park, Imperial College London. The samples were then analyzed independently in the two laboratories. The DNA from specimen 1 is available at Silwood Park DNA & Tissue Bank (CITES GB038) under accession VS8956-20002085971; DNA from specimen 2 is available at Hopkins Marine Station, Stanford University under accession 020002232485. For specimen 1, genomic DNA was extracted using Qiagen’s Blood & Tissue Kit and was sequenced using an Illumina HiSeq. The mitochondrial genome sequence was assembled using ABySS v2.0.2 (Jackman et al. 2017) and GapCloser v1.12 (Luo et al. 2012). For specimen 2, the DNA was extracted and sequenced using the Oxford Nanopore Technologies’ MinION sequencer following Johri et al. (Johri et al. 2019). The MitoFish mitoannotator (Iwasaki et al. 2013) was used to annotate the sequences, and these were aligned against one another and mitogenomes from other Carcharhinid species using MUSCLE (Edgar 2004) within Geneious Prime (v2019.0.4). A phylogenetic tree was produced in Geneious Prime using MrBayes (Huelsenbeck and Ronquist 2001; Ronquist et al. 2012) plugin (v.3.2.6, substitution model: HKY85, burn-in length: 100,000) using the gray bamboo shark (*Chiloscyllium griseum;* NC_017882) and scalloped hammerhead shark (*Sphyrna lewini;* NC_022679) as outgroups.

The complete mitochondrial genomes are 16,705 bp (specimen 1) and 16,706 bp (specimen 2) in length. Each contains two rRNAs, 22 tRNAs, 13 protein-coding genes and a non-coding control region. The nucleotide base composition is identical with 31.5% A, 25.2% C, 13.2% G and 30.1% T, the overall GC content is 38.4%. The two sequences have 16,682 identical sites (99.9% pairwise identity). The differences include one base addition and 23 substitutions. Four substitutions result in a change to the amino acid sequence of COI. These differences could be due to the sequencing methods or represent evidence of population structure within the species in BIOT despite high spatial connectivity across the territory (Carlisle et al. 2019).

**Figure 1.**
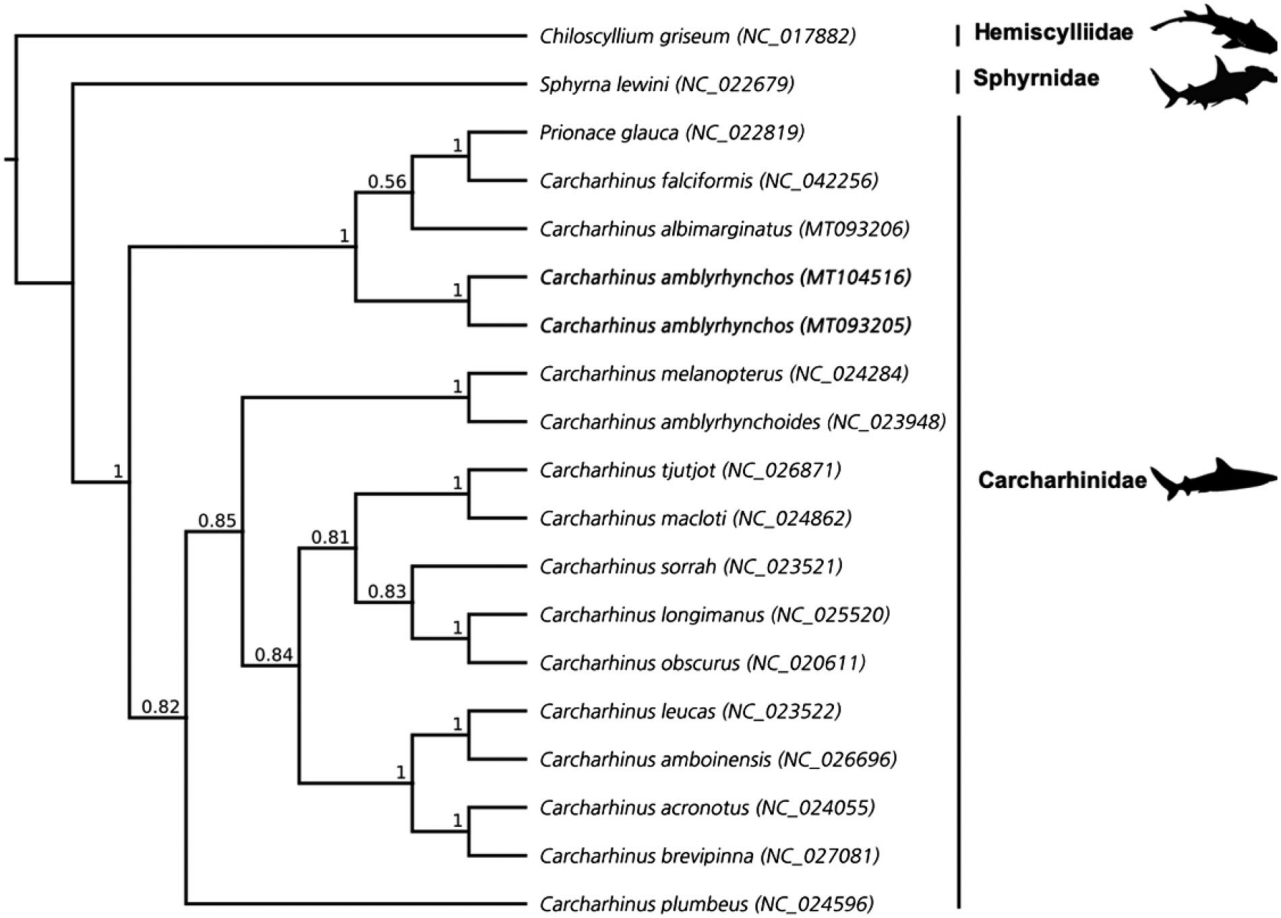
Cladogram showing the phylogenetic relationship of species with complete mitogenome sequences in the genus *Carcharhinus* including *Prionace glauca*, with the scalloped hammerhead shark (*Sphyrna lewini*) and gray bamboo shark (*Chiliscyllium griseum*) as outgroups. The new sequences for the gray reef shark (*Carcharhinus amblyrhynchos*) are in bold. Families are indicated by vertical lines and represented by silhouettes accessed from PhyloPic (phylopic.org). Values at each node represent the Bayesian posterior probability at each node, GenBank accession numbers for each sequence are in brackets.

Whilst the fine-scale phylogenetic relationships within Carcharhinidae remain unresolved (Naylor et al. 2012), the tree (Figure 1) supports the placement of *C. amblyrhynchos* in a clade with *C. albimarginatus, C. falciformis,* and *Prionace glauca.* The low posterior probability that supports the placement of *P. glauca* with *C. albimarginatus* and *C. falciformis* suggests that further work is required to fully resolve the tree. However, the high support for deeper clades within *Carcharhinus* adds to calls for a taxonomic revision of *P. glauca* (Naylor et al. 2012; Johri et al. 2019). The new mitochondrial genomes presented here will aid in conservation genetics, environmental DNA and population studies as researchers move toward assessing populations using genome sequences.

## Data Availability

The data that support the findings of this study are openly available in GenBank at https://www.ncbi.nlm.nih.gov/nuccore/MT093205 reference number MT093205.1 and https://www.ncbi.nlm.nih.gov/nuccore/MT104515 reference number MT104515.1.
